# COVID-19: it is time to balance infection management and person-centered care to maintain mental health of people living in German nursing homes

**DOI:** 10.1017/S1041610220000897

**Published:** 2020-05-12

**Authors:** Martin N. Dichter, Marco Sander, Swantje Seismann-Petersen, Sascha Köpke

**Affiliations:** Institute of Nursing Science, Medical Faculty, University of Cologne, Cologne, Germany

## COVID-19 in Germany

Globally, the number of people infected with COVID-19 is still increasing. With currently more than 160,000 confirmed cases, Germany ranks sixth worldwide behind the USA, Spain, Italy, France, and the United Kingdom (John Hopkins University, 2020). Severe courses of COVID-19 with increased mortality are particularly evident in elderly people and those with chronic diseases like hypertension, diabetes, or coronary heart disease (Wang *et al.*, 2020a; Zhou *et al.*, 2020). People living in nursing homes seem to be at a particularly high risk of dying from COVID-19. Between 33% and 65% of all people who died from COVID-19 were nursing home residents in various European countries (Comas-Herrera *et al.*, 2020).

Although the validity of these early data must be questioned, it does indicate that nursing home residents are a particularly vulnerable group. In Germany, approximately 818,000 people live in 14,500 nursing homes (Bundesamt, 2017). Nursing home-specific data for COVID-19 infections and deaths are not available, as in Germany data are reported for nursing homes together with other institutions such as homes for disabled and dependent persons, shelters for homeless people, collective accommodation for asylum seekers, and prisons. In these populations, available data show *N* = 10,040 COVID-19 cases, representing approximately 7% of all infections in Germany. Significantly, 1,806 (18%) of these people died, which is 30% of all COVID-19 related deaths (Robert Koch Institut, 2020).

## Vulnerability of people living in nursing homes to infectious diseases

Older people living in nursing homes are particularly vulnerable to infectious diseases such as COVID-19. The reasons for this are compromised physiological barriers (e.g. skin breakdown, use of catheters), immunosuppression, malnutrition, dehydration, functional impairments (e.g. incontinence, immobility) (Stone, 2017), hearing and vision impairment and cognitive impairment. Moreover, there are risk factors at the institutional level that favor infectious diseases in nursing homes. These factors are staff shortage, sharing bathroom facilities, mutual social activities, and low preparedness for infection control (Davidson and Szanton, 2020). In addition, in Germany as in many other countries, there is a shortage of urgently needed personal protective equipment for nursing home staff, including masks and gowns.

## Current consequences for nursing home care in Germany

Due to this particular vulnerability and the simultaneously increased lethality of COVID-19 for old and chronically ill people, the recently introduced infection control measures for German nursing homes are particularly restrictive. The introduction of regional, state, and federal regulations to protect residents from an infection has led to bans on leaving and visiting nursing homes (State Government of North Rhine-Westphalia, 2020). Since mid-March, the doors have been closed to relatives and residents alike. This means that relatives or friends cannot visit residents, and residents cannot leave the nursing home property. With few exceptions, these restrictive rules also apply to health professionals, for example, physicians, physiotherapists or speech therapists, and service providers such as hairdressers who are not part of the regular nursing home staff in Germany. Moreover, group-based social activities are often cancelled completely due to the inability to guarantee a safety distance of 1.5–2 m between persons according to the size of the room, the cognitive abilities of the residents, and/or residents’ behavior (e.g. wandering). This also applies to meals that were previously shared together.

Depending on the federal state, further restrictions such as admission bans have been implemented. In North Rhine-Westphalia, Germany’s most populous state, new residents can still be admitted, but nursing homes have to provide a quarantine and isolation unit in addition to a regular care unit. These units must be separated both spatially and in terms of personnel, irrespective of the basic unit and personnel team structure of the facility. People without a suspected or confirmed SARS-CoV-2 infection are cared for in the regular care unit. Residents recently admitted from hospital or home must be cared for in the quarantine unit for at least 14 days. Residents with a confirmed COVID-19 infection have to be cared for in the isolation unit (Ministry of Labour Health and Social Affairs Northrhine-Westphalia, 2020).

In cases where people with cognitive impairment (e.g. dementia) and challenging behavior need to be isolated, this is usually done in a separate unit or room (e.g. in the rooms of a closed day care facility which can be a subsidiary facility of a nursing home) and often with a caregiver who is then responsible only for that particular resident. In times of staff shortages, such cases are a great challenge for nursing home care.

Nursing homes in Germany are usually well occupied, although there are still some facilities that have twin rooms. Therefore, these requirements can only be implemented by separating residents from everything they are used to: their personal rooms, trusted cohabitants, trusted caregivers as well as their normal daily structure and activities.

The ban on visits for nursing homes represents a serious restriction on residents’ rights to self-determination. Thus, it is currently not possible to have personal contact with relatives, friends, and spouses who do not live in the same nursing home. Possibilities for sharing personal fears (e.g. concerning the current situation) and worries and for talking about everyday topics are reduced to telephone or video calls. Apart from direct contacts, any support that residents have received previously from relatives and friends is no longer possible, for example, company during walks outside, reading aloud newspaper articles or books, purchasing food and drinks as well as physical closeness (e.g. a hug, holding of hands). Assistance at mealtimes, skin and hair care or, for instance, massaging an aching shoulder by relatives is currently not allowed. In addition, relatives and friends are missed as advocates, translators, and communicators of residents’ needs.

This is particularly dramatic as the nursing home residents often have a limited life expectancy; approximately 20% die within 12 months (Vetrano *et al.*, 2018). Moreover, between 70% and 80% of nursing home residents are affected by dementia (Helvik *et al.*, 2015; Rothgang, 2010), a life-limiting disease requiring palliative care (van der Steen *et al.*, 2014). The dramatic consequences of the current visiting restrictions are highlighted by the fact that relatives and friends can no longer attend even dying residents.

The number of people who feel lonely and depressed is already very high under normal conditions with 30% of nursing home residents with acute depression (Kramer *et al.*, 2009) and 50–55% experiencing loneliness (Drageset *et al.*, 2011; Nyqvist *et al.*, 2013). Loneliness is associated with an array of health problems such as hypertension, cardiovascular disease, cognitive decline, depression, and early mortality (Gerst-Emerson and Jayawardhana, 2015). Moreover, social isolation is associated with an increased memory decline (Read *et al.*, 2020). It can therefore be assumed that the current infection control measures clearly have negative consequences, especially for a resident’s mental health status as a result of social isolation.

## Person-centered care during COVID-19 pandemic conditions

If on one side of a care continuum there are restrictions and social isolation measures, on the other side of the continuum there is person-centered care. This can be defined as a holistic approach to care in a respectful and individualized manner, including how care is negotiated, and offering a choice through a therapeutic relationship. Here, residents are empowered to be involved in health decisions as desired by the individual (Morgan and Yoder, 2012).

In spite of the current restrictive infection control measures, the principles of person-centered care must be implemented in nursing home care. Therefore, infection management and person-centered care have to be weighed carefully in order to maintain the residents’ social participation, mental health, and quality of life.

Nursing home residents need comprehensive information about the COVID-19 pandemic and the resulting infection control measures. Based on this information, residents and caregivers have to negotiate the implementation of infection management measures and to deal with the need for the provision of person-centered care under the current circumstances. Moreover, several additional interventions are needed in order to reduce social isolation and its negative consequences for residents. Residents must have assistance when making telephone and/or video calls with their relatives and friends. Individual social activities must be offered more often by caregivers, and group-based social activities should be provided as long as the defined safety distance between the participants can be complied with (in Germany 1.5–2m). If room sizes are limited, group sizes must be reduced accordingly. Despite limited personnel resources, residents must be given the opportunity to walk or spend time outdoors and there must be opportunities for relatives and residents to see and talk to each other, in compliance with the infection management regulations.

Nurses must focus especially on detecting symptoms of COVID-19 but also pay attention to the mental health problems of the residents. For this, it is essential to have interaction with the residents and to actively manage the care relationship in spite of face masks and perhaps other personal protective equipment. A reduced contact time between caregivers and residents, as is known, for example for contact isolated intensive care patients (Kirkland and Weinstein, 1999), must be avoided. If mental health problems are identified, interventions to provide mental health and psychosocial support must be examined and implemented (Wang *et al.*, 2020b).

In addition, personal end-of-life care must be guaranteed. For this purpose, personal protective equipment and comprehensive information, if possible for the resident and his/her relatives and friends, are necessary.

## Outlook and suggestions for the future

The reported figures from official statistics as well as recent research show the widespread transmission of SARS-CoV-2 and the high lethality of COVID-19 in nursing home residents (Arons *et al.*, 2020). Moreover, more than half of the residents with a positive COVID-19 diagnosis are asymptomatic at the time of testing. These asymptomatic cases very likely contribute to the transmission of the virus. Therefore, broad and frequent testing of all nursing home residents and their caregivers is recommended (Arons *et al.*, 2020; Centers for Disease Control and Prevention, 2020). In addition, sufficient personal protective equipment is needed for nursing home staff and also for relatives and friends of residents. The measures seem to be of utmost importance as a basis to reduce the spreading of the SARS-CoV-2 virus and to reopen nursing homes for visits by residents’ relatives and friends.

Sensible intermediate steps are needed to allow for the implementation of these measures. Under the impact of the infection management regulations and their serious consequences for the social participation of nursing home residents, a group of researchers and practitioners are currently working on an expert-based guideline for Germany coordinated by the German Society of Nursing Science (DGP). This guideline is intended to offer recommendations for nursing homes in order to enable a maximum of social participation and quality of life even under COVID-19 pandemic conditions (AWMF, 2020).

We are writing this commentary at a moment, when the impact of the COVID-19 pandemic develops around us daily. Just now, we can only rely on anecdotal knowledge about the current care situation in nursing homes. Empirical studies are urgently needed to learn more about the situation of nursing home residents, their caregivers and relatives for this and especially for future pandemics. Moreover, we agree with the position of the German Network for Evidence-based Medicine that have recently called for a clinical–epidemiological database to be created by systematic testing, systematic documentation, and – most importantly – meaningful research concerning care models for pandemic situations that allow for a maximum of protection with a minimum of freedom restrictions (German network for evidence-based medicine, 2020).
